# EANM practical guidance on uncertainty analysis for molecular radiotherapy absorbed dose calculations

**DOI:** 10.1007/s00259-018-4136-7

**Published:** 2018-09-14

**Authors:** Jonathan I. Gear, Maurice G. Cox, Johan Gustafsson, Katarina Sjögreen Gleisner, Iain Murray, Gerhard Glatting, Mark Konijnenberg, Glenn D. Flux

**Affiliations:** 1 The Royal Marsden NHS Foundation Trust & Institute of Cancer Research, Downs Road, Sutton, SM2 5PT UK; 2 National Physical Laboratory, Teddington, TW11 0LW UK; 3 Department of Medical Radiation Physics, Clinical Sciences Lund, Lund University, Lund, Sweden; 4 Medical Radiation Physics, Department of Nuclear Medicine, Ulm University, Ulm, Germany; 5 Nuclear Medicine, Erasmus MC, Rotterdam, The Netherlands

**Keywords:** Dosimetry, Uncertainty analysis

## Abstract

A framework is proposed for modelling the uncertainty in the measurement processes constituting the dosimetry chain that are involved in internal absorbed dose calculations. The starting point is the basic model for absorbed dose in a site of interest as the product of the cumulated activity and a dose factor. In turn, the cumulated activity is given by the area under a time–activity curve derived from a time sequence of activity values. Each activity value is obtained in terms of a count rate, a calibration factor and a recovery coefficient (a correction for partial volume effects). The method to determine the recovery coefficient and the dose factor, both of which are dependent on the size of the volume of interest (VOI), are described. Consideration is given to propagating estimates of the quantities concerned and their associated uncertainties through the dosimetry chain to obtain an estimate of mean absorbed dose in the VOI and its associated uncertainty. This approach is demonstrated in a clinical example.

## Introduction

Internal dosimetry following the administration of radiolabelled pharmaceuticals for diagnostic and therapeutic purposes is a prerequisite for radiation protection, imaging optimization, patient-specific administrations and treatment planning. The medical internal radiation dose (MIRD) schema [1] has become the most widely accepted formalism for internal dose calculations. The general approach in medicine to determine the validity of a measurement is to compare the accuracy and precision against a “gold standard” measurement. To date, investigations of uncertainties for internal dosimetry have mainly used phantoms or simulated data [2–4] as the gold standard comparison. However, due to the diversity of dosimetry data, a subset of phantom experiments cannot necessarily validate the accuracy of measurements made for an in vitro population. It is therefore more appropriate to express the accuracy of a result by characterizing the uncertainty. This involves identification of the major processes and variables within the dose calculation and evaluation of their potential effect on the measurement. An uncertainty estimate should address all systematic and random sources of error and characterize the range of values within which the measured value can be said to lie with a specified level of confidence. The general relevance of performing and providing uncertainty information has been discussed in previous guidelines [5]. Flux et al. [6] provided a method to determine the uncertainty of whole-body absorbed doses calculated from external probe measurement data. Whilst whole-body dosimetry is used to predict toxicity in some procedures [7, 8], organ and tumour dosimetry are required for treatment planning and in cases where haematotoxicity is not the limiting factor for treatment tolerance.

Specific aspects of uncertainty within the dosimetry chain have been addressed, including the selection of measured time points, [9, 10], the chosen fit function [11, 12] and uncertainty of model parameters. A comprehensive analysis of propagation of every aspect of the dosimetry calculation chain has yet to be obtained.

Gustafsson et al. [13] adopted a Monte Carlo (MC) approach to investigate the propagation of uncertainties to obtain an uncertainty in estimated kidney absorbed dose in ^177^Lu-DOTATATE therapy, using simulated gamma-camera images of anthropomorphic computer phantoms. In principle, this approach allows all aspects of the dosimetry process to be taken into account, but the need for multiple samplings from the assigned probability distributions for the quantities involved makes it computationally intensive, and its use for uncertainty assessment in an individual patient basis is challenging.

The Joint Committee for Guides in Metrology (JCGM) Guide to the Expression of Uncertainty in Measurement (GUM) [14] provides a generalized schema for propagating uncertainties. This EANM Dosimetry Committee guidance document provides recommendations on how to determine uncertainties for dosimetry calculations and apply the law of propagation of uncertainty (LPU) given in the GUM to the MIRD schema. This guidance document is presented in the form of an uncertainty propagation schema, and the recommendations are designed to be implemented with the resources available in all nuclear medicine departments offering radionuclide therapy, and are presented using terminology and nomenclature that adhere as far as possible to the GUM.

The uncertainty propagation schema examines each step of the absorbed dose calculation to estimate the standard uncertainty in the mean absorbed dose measured at the organ or tumour level using SPECT imaging. The examples given have been simplified and concentrate only on the mean absorbed dose to a target. However, the approach can be used in different scenarios and expanded to include more complex dose calculations, including cross-dose and multiexponential time–activity curves (TACs). Similarly, aspects of the methodologies described can be implemented in different applications of dosimetry such as those utilizing a hybrid imaging approach or those used to generate 3D dose maps.

## Theory

### The law of propagation of uncertainty

A generic multivariate measurement model is:

1 $$ \boldsymbol{Y}=\boldsymbol{f}\left(\boldsymbol{X}\right), $$

where

2 $$ \boldsymbol{X}={\left[{X}_1,\dots, {X}_n\right]}^{\top } $$

is a vector of *n* generic input quantities *X*_1_, …, *X*_*n*_ and

3 $$ \boldsymbol{Y}={\left[{Y}_1,\dots, {Y}_m\right]}^{\top } $$

is a vector measurand of *m* output quantities *Y*_1_, …, *Y*_*m*_. GUM Supplement 2 [15] gives a generalization of the LPU:

4 $$ {\boldsymbol{V}}_{\boldsymbol{y}}={\boldsymbol{G}}_{\boldsymbol{x}}{\boldsymbol{V}}_{\boldsymbol{x}}{\boldsymbol{G}}_{\boldsymbol{x}}^{\top } $$

where ***V***_***y***_ is the output covariance matrix associated with ***y*** (the estimate of ***Y***). ***V***_***x***_ is the input covariance matrix

5 $$ {\boldsymbol{V}}_{\boldsymbol{x}}=\left[\begin{array}{ccc}{u}^2\left({x}_1\right)& \dots & u\left({x}_1,{x}_n\right)\\ {}\vdots & \ddots & \vdots \\ {}u\left({x}_n,{x}_1\right)& \dots & {u}^2\left({x}_n\right)\end{array}\right] $$

associated with

6 $$ \boldsymbol{x}={\left[{x}_1,\dots, {x}_n\right]}^{\top }, $$

the estimate of *X*, and *G*_*x*_ is the sensitivity matrix associated with *x*, defined as:

7 $$ {\boldsymbol{G}}_{\boldsymbol{x}}=\left[\begin{array}{ccc}\frac{\partial {f}_1}{\partial {x}_1}& \dots & \frac{\partial {f}_1}{\partial {x}_n}\\ {}\vdots & \ddots & \vdots \\ {}\frac{\partial {f}_m}{\partial {x}_n}& \dots & \frac{\partial {f}_m}{\partial {x}_n}\end{array}\right], $$

where ∂*f*_*i*_/∂*x*_*j*_ denotes ∂*f*_*i*_/∂*X*_*j*_ evaluated at *X* = *x*. Element *u*(*x*_*i*_, *x*_*j*_) of *V*_*x*_ is the covarience associated with *x*_*i*_ and *x*_*j*_, and *u*(*x*_*i*_, *x*_*i*_) is equal to *u*^2^(*x*_*i*_), the squared uncertainty associated with *x*_*i*_. *x* and *V*_*x*_ are obtained from available knowledge, whether statistical (for example, repeated observations) or nonstatistical (for example, expert judgment), about the input quantities.

For a generic scalar measurement model, Eq. 1 becomes *Y* = *f*(*X*), where *Y* is a scalar quantity and *f* is a scalar function. Propagation of uncertainty for the estimate *y* of *Y* can be achieved using the matrix form of the LPU [15]:

8 $$ {u}^2(y)={\boldsymbol{g}}_{\boldsymbol{x}}^{\top }{\boldsymbol{V}}_{\boldsymbol{x}}{\boldsymbol{g}}_{\boldsymbol{x}}, $$

where *u*^2^(*y*) represents the variance (squared standard uncertainty) associated with the estimate *y*, and

9 $$ {\boldsymbol{g}}_x=\left[\begin{array}{c}\frac{\partial f}{\partial {x}_1}\\ {}\vdots \\ {}\frac{\partial f}{\partial {x}_n}\end{array}\right]\kern0.5em $$

is the gradient matrix in which the *i*th element denotes the partial derivative of *f* with respect to the quantity *X*_*i*_ evaluated at *x*.

For a two-variable function, *Y* = *f*(*X*_1_, *X*_2_), Eq. 8 can be expanded to give:

10 $$ {u}^2(y)={\left(\frac{\partial f}{\partial {x}_1}\right)}^2{u}^2\left({x}_1\right)+{\left(\frac{\partial f}{\partial {x}_2}\right)}^2{u}^2\left({x}_2\right)+2\frac{\partial f}{\partial {x}_1}\frac{\partial f}{\partial {x}_2}u\left({x}_1,{x}_2\right). $$

For a two-variable multiplicative function, *Y* = *X*_1_*X*_2_, Eq. 10 can be written in the form:

11 $$ {\left[\frac{u(y)}{y}\right]}^2={\left[\frac{u\left({x}_1\right)}{x_1}\right]}^2+{\left[\frac{u\left({x}_2\right)}{x_2}\right]}^2+2\frac{u\left({x}_1,{x}_2\right)\ }{x_1{x}_2}. $$

If the two variables *X*_1_ and *X*_2_ are mutually independent, the covariance term of Eq. 11 is zero, and therefore the standard fractional uncertainties *u*(*x*_1_)/*x*_1_ and *u*(*x*_2_)/*x*_2_ are simply added in quadrature.

### Absorbed dose

For situations where the target volume is the source activity volume and the contribution of absorbed dose from other target organs can be considered negligible, a simplified form of the MIRD equation can be used in which mean absorbed dose $\overline{D}$ is expressed as the product of the cumulated activity $\overset{\sim }{A}$ and the S-factor (sometimes called the dose factor) *S*:

12 $$ \overline{D}=\overset{\sim }{A}S. $$

Following the above notation,$\overline{D}$ is written as $\overline{D}=f\left(\overset{\sim }{A},S\right)=\overset{\sim }{A}S$, and the standard uncertainty $u\left(\overline{D}\right)$ is evaluated at estimates of $\overset{\sim }{A}$ and *S* according to Eq. 11:

13 $$ {\left[\frac{u\left(\overline{D}\right)}{\overline{D}}\right]}^2={\left[\frac{u\left(\overset{\sim }{A}\right)}{\overset{\sim }{A}}\right]}^2+{\left[\frac{u(S)}{S}\right]}^2+2\frac{u\left(\overset{\sim }{A},S\right)\ }{\overset{\sim }{A}S}. $$

It follows that the standard uncertainties $u\left(\overset{\sim }{A}\right)$ and *u*(*S*) and the covariance $u\left(\overset{\sim }{A},S\right)$ are needed to obtain the standard uncertainty $u\left(\overline{D}\right)$ associated with $\overline{D}$.

For the general form of the MIRD equation with meaningful contributions outside the target volume (cross-dose), uncertainties and covariances associated with additional quantities of the form $\overset{\sim }{A}$ and *S* should also be considered.

**Fig. 1:**
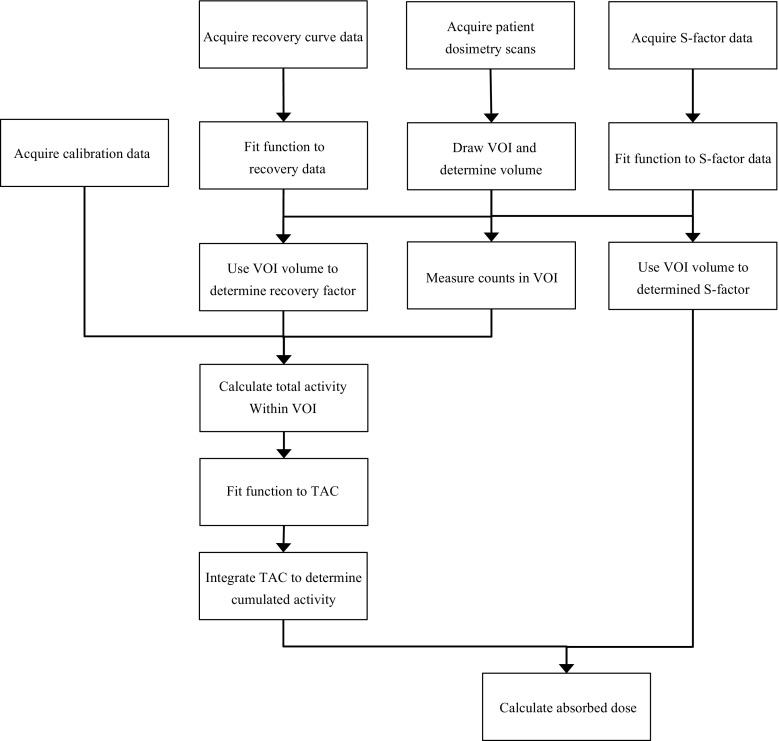
Flow diagram showing chronological sequence of the dosimetry schema demonstrating how uncertainty can propagate between each step

The need for the covariance term of Eq. 13 may not be obvious on first inspection as calculations of $\overset{\sim }{A}$ and *S* are often considered separately, that is, one is derived from scintigraphy data and the other from simulations. However, as can be seen from Fig. 1 (a flow diagram of a typical dosimetry protocol), determination of $\overset{\sim }{A}$ and *S* both rely on a measurement of volume, and therefore a covariance exists between the two parameters. This EANM Dosimetry Committee guidance document describes how the uncertainty in the volume measurement and other confounding factors within the dosimetry protocol can be propagated to estimate an overall uncertainty in absorbed dose.

### Volume uncertainty

The volume or mass of an organ or tumour is generally obtained from a volume of interest (VOI) outlined on anatomical or functional imaging data. It is therefore possible to estimate the outlining accuracy by considering factors that affect delineation. The method used will depend largely on the information and resources available at the time of outlining and the method employed by the operator or operators to define the VOI. The following concerns an outline drawn manually by a single operator across all images that comprise the dosimetry dataset.

#### Operator variability

For any given dosimetry dataset a number of independent VOIs could be drawn by different operators. Ideally, an average VOI boundary of volume $\overline{v}$ would then be generated and used in the calculation of absorbed dose. In practice, an average VOI boundary cannot be generated and an absorbed dose calculation is based upon the VOI drawn by a single operator. An alternative approach is therefore to estimate operator variability using historical datasets. In this case outlines previously generated by *M* different operators for *N* similar VOIs are used. The assumption made is that the VOIs are sufficiently similar with respect to the scanning modality and volume geometry that the fractional uncertainty is the same across all datasets. The VOI outlined by the current operator is then regarded as a random VOI drawn from the populations of outlines represented by the historical data. The standard uncertainty *u*(*v*) associated with the drawn volume is then expressed as:

14 $$ {\left(\frac{u(v)}{v}\right)}^2=\frac{1}{N^2}\sum \limits_{n=1}^N{\left(\frac{s\left({v}_{{\mathrm{hist}}_i}\right)}{v_{{\mathrm{hist}}_i}}\right)}^2, $$

where${v}_{{\mathrm{hist}}_i}$ is the average of *M* operator volumes for the historical dataset *i*, and $s\left({v}_{{\mathrm{hist}}_i}\right)$is the standard deviation of the historical dataset *I*,

15 $$ {s}^2\left({v}_{{\mathrm{hist}}_i}\right)=\frac{\sum_{m=1}^M{\left({v}_m-{v}_{{\mathrm{hist}}_i}\right)}^2}{M-1}. $$

The historical datasets should be carefully chosen to match the current study, as differences could lead to an inaccurate estimate of the final standard deviation.

#### Analytical approach

When historical outlines are not available, it is possible to use an analytical method to determine uncertainty. This approach provides an estimate of the most significant contributions to the uncertainty in the outlining process but is not necessarily exhaustive.

**Fig. 2:**
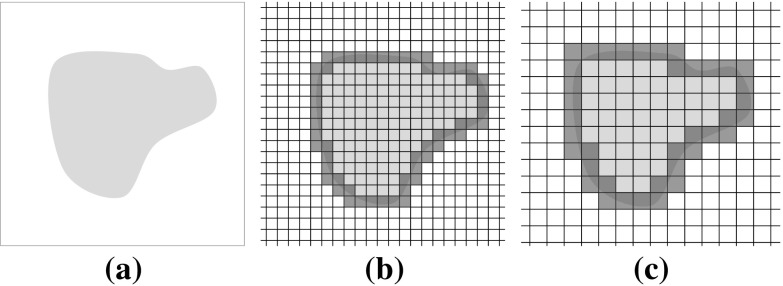
Uncertainty in outline definition for different voxel sizes

Given that any outlined VOI will be digitized into voxels, the extent of the VOI will be defined, approximately, by the subset of voxels through which the boundary of the VOI passes. The uncertainty of the outlined volume will hence depend on voxel size. Figure 2 shows an outline (Fig. 2a) and the effect of different voxel sizes (Fig. 2b, c).

**Fig. 3:**
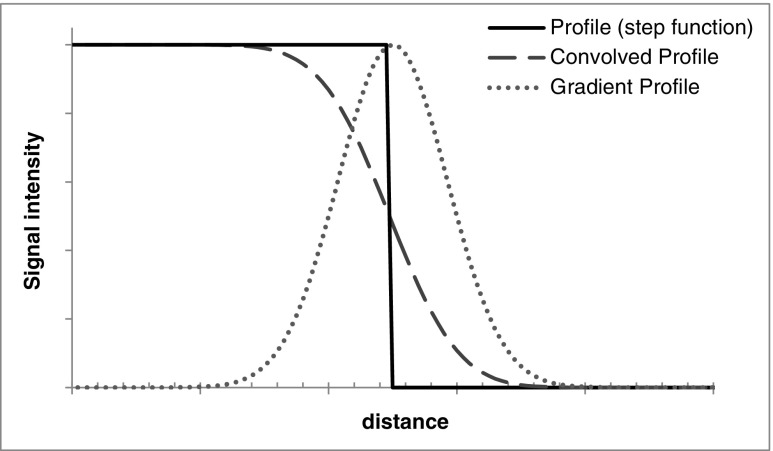
Signal intensity profiles demonstrating that the gradient of a Gaussian blurred function can be described by the Gaussian function

The mass of a spherical volume may be obtained from an estimate of the diameter of the volume, where the diameter *d* is measured as the distance between two extreme points, *P*_*I*_ and *P*_*j*_, the locations of which can be determined within one voxel dimension (Fig. 3).

Evaluation of the standard uncertainty associated with *d* can be considered as type B (nonstatistical), as in the GUM [14]. Given that there is no specific knowledge about the location of point *P*_*i*_ on the boundary, other than that it lies within the appropriate boundary voxel, there is a uniform distribution of possible values with variance associated with the mean value given by formula 7 in [14]:

16 $$ {u}^2\left({P}_i\right)=\frac{a^2}{12}\kern0.5em $$

where *a* is one voxel width and *u*(*P*_*i*_) is to be interpreted as the standard uncertainty associated with *P*_*i*_ when measured on the diametric line between *P*_*i*_ and the centre of the sphere. As diametric measurement is the distance between two extreme points, application of the variant of LPU [14] (formula 11a in the GUM) yields (assuming no correlation) the standard uncertainty *u*_vox_(*d*) associated with diameter *d* due to voxelization:

17 $$ {u}_{\mathrm{vox}}^2(d)={u}^2\left({P}_i-{P}_j\right)=\frac{a^2}{6}. $$

With hybrid imaging it is often possible to use morphological information from CT imaging to aid functional VOI delineation. In this situation the VOI is drawn on the CT dataset and copied to the registered SPECT image. The coordinates of the original boundary will therefore be rounded to the nearest voxel coordinates of the SPECT image. Hence, the SPECT voxel size should be used in Eq. 17.

For many scintigraphic imaging processes the defined voxel size is less than the spatial resolution of the system and therefore the use of Eq. 17 would result in underestimation of the actual uncertainty when the VOI is drawn directly on the SPECT image. To provide a more reliable uncertainty the spatial resolution of the image system must also be considered. Consider a profile through an object approximated as a step function convolved with a Gaussian point spread function (PSF) with the full-width at half-maximum (FWHM) equal to the spatial resolution of the imaging system. The uncertainty in edge definition can be described by the gradient of the convolved step function, where the gradient profile is equal to the Gaussian PSF with standard deviation $\sigma =\frac{\mathrm{FWHM}}{2\sqrt{2\ln 2}}$. As the diametric measurement is the distance between the boundary locations on the profile, application of the variant of LPU [14] (GUM formula 11a) yields the standard uncertainty *u*_res_(*d*) associated with diameter *d* due to spatial resolution:

18 $$ {u}_{\mathrm{res}}^2(d)=2{\sigma}^2={\left(\frac{\mathrm{FWHM}}{2\sqrt{\ln 2}}\right)}^2. $$

For situations where both voxelization and resolution contribute significantly to diametric uncertainty (such as outlining directly on a SPECT image), Eqs. 17 and 18 are summed to give the combined uncertainty associated with *d*:

19 $$ {u}^2(d)={u}_{\mathrm{vox}}^2(d)+{u}_{\mathrm{res}}^2(d)=\frac{a^2}{6}+\frac{{\left(\mathrm{FWHM}\right)}^2}{4\ln 2}. $$

In practice the diameter is not measured and only the volume is reported. However, conceptually the volume is determined through an infinite number of diametric measurements, and the mean diameter of the VOI can therefore be considered. The standard uncertainty *u*(*d*) translates into a standard uncertainty *u*(*v*) associated with the volume *v* delineated by the outline. Hence, for some positive constant *k*,

20 $$ v=k{d}^3. $$

The application of the variant of LPU [14] (GUM formula 12) yields a relationship between the relative volume and diametric uncertainties due to voxelization and resolution:

21 $$ {\left[\frac{u(v)}{v}\right]}^2={\left[3\frac{u(d)}{d}\right]}^2={\left[3\frac{u_{\mathrm{vox}}(d)}{d}\right]}^2+{\left[3\frac{u_{\mathrm{res}}(d)}{d}\right]}^2. $$

Hence, a fractional standard uncertainty associated with a volume is three times the fractional standard uncertainty associated with the mean diameter of that volume.

### Count rate

The total reconstructed count rate, *C*, within a VOI depends on the VOI delineation, and can be described as a function of volume. Propagation of volume uncertainty into the measurement of count rate is therefore required. As no prior knowledge of the count distribution is generally available, the variation in *C* within the VOI must be approximated.

A uniformly distributed spherical count rate density *H*(*ρ*) with volume *v*_true_ of radius *r*, with a total emission count rate of *C*_total_, can be described in spherical coordinates as:

22 $$ H\left(\rho \right)=\left\{\begin{array}{c}\frac{C_{\mathrm{total}}}{v_{\mathrm{true}}},\kern0.5em \rho <r,\\ {}0,\kern4.25em \rho \ge r,\end{array}\right. $$

where *ρ* is the radial distance from the centre of the sphere.

Due to the spatial limitations of the measuring system the apparent density is described as the spherical volume convolved with a 3D Gaussian function [16]:

23 $$ G\left(\rho \right)=\frac{1}{{\left[\sigma \sqrt{2\pi}\right]}^3}{e}^{-\frac{\rho^2}{2{\sigma}^2}}, $$

where *σ* is the measured standard deviation describing the width of the 3D Gaussian function. Therefore, an observed count rate density distribution can be described as:

24 $$ F\left(\rho \right)=H\left(\rho \right)\ast G\left(\rho \right), $$

where ∗ denotes convolution in three dimensions, which can be determined analytically [17] and re-expressed as:

25 $$ F\left(\rho \right)=\frac{C_{\mathrm{total}}}{2{v}_{\mathrm{true}}}\left[\operatorname{erf}\left(\frac{r-\rho }{\sigma \sqrt{2}}\right)+\operatorname{erf}\left(\frac{r+\rho }{\sigma \sqrt{2}}\right)-\frac{2\sigma }{\rho \sqrt{2\pi }}{e}^{-\left(\frac{r^2+{\rho}^2}{2{\sigma}^2}\right)}\left[{e}^{\left(\frac{r\times \rho }{\sigma^2}\right)}-{e}^{-\left(\frac{r\times \rho }{\sigma^2}\right)}\right]\right] $$

**Fig. 4:**
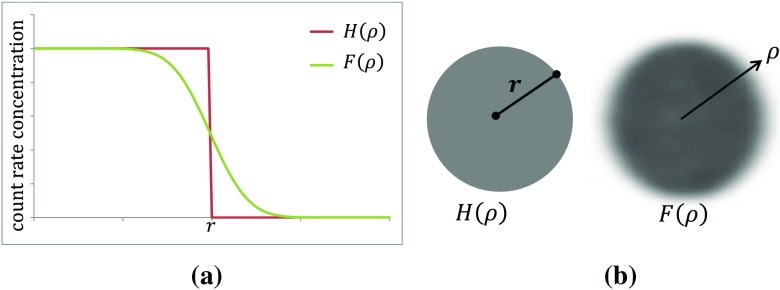
**a** Count density as function of radius for a spherical object with true radius *r* for a system with ideal resolution (*red step function*) and realistic system (*green curve*). **b** Two-dimensional image planes through the three-dimensional functions *H*(*ρ*) and *F*(*ρ*) (see text)

The function *F*(*ρ*) and that of perfect resolution *H*(*ρ*) are shown in Fig. 4a; images of these distributions are shown in Fig. 4b.

**Fig. 5:**
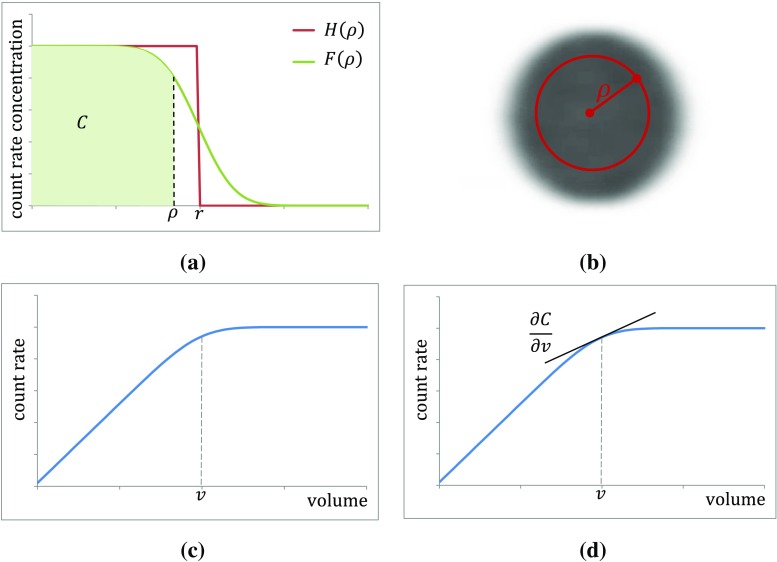
**a** Count density as function of radius showing observed counts within a VOI of radius *ρ* (*green shaded area*). **b** Two-dimensional image plane with the VOI outlined with radius ***ρ*** (*red line*). **c** Count rate as a function of VOI volume *v* corresponding to a particular choice of radius *ρ*. **d** Gradient of the count rate with respect to the VOI volume *v*

Using Eq. 25, the count rate *C* measured within a VOI of volume *v* and radius *ρ* can be expressed as:

26 $$ C={\int}_0^vF\left(\rho \right)\mathrm{d}v, $$

as shown in Fig. 5, where *C* is described as the area under the curve. A plot of this function with increasing *ρ* and *v* is given in Fig. 5a.

As there is an uncertainty associated with the drawn VOI boundary, the standard uncertainty *u*(*C*) associated with *C* is obtained using the gradient of *C* at *v* (Fig. 5d) and the volume standard uncertainty *u*(*v*):

27 $$ u(C)=\frac{\partial C}{\partial v}u(v)=F\left(\rho \right)u(v). $$

For a VOI of volume *v*, where the radius *ρ* = *r*, the expression for *F*(*ρ*) as given by Eq. 25 can be substituted into Eq. 27:

28 $$ \frac{\partial C}{\partial v}u(v)=\frac{C_{\mathrm{total}}\varphi }{2v}u(v), $$

where

29 $$ \varphi =\operatorname{erf}\left(\frac{2r}{\sigma \sqrt{2}}\right)-\frac{2\sigma }{r\sqrt{2\pi }}\left[1-{e}^{-\left(\frac{2{r}^2}{\sigma^2}\right)}\right]. $$

The ratio of the total emission count rate from a source to the count rate measured within a VOI defining the physical boundary is referred to as the recovery coefficient [18] (see section Recovery coefficient):

30 $$ R=\frac{C}{C_{\mathrm{total}}}. $$

Therefore, the standard uncertainty *u*(*C*) associated with *C* can be rewritten as:

31 $$ \frac{u(C)}{C}=\frac{\varphi }{2R}\frac{u(v)}{v}. $$

### Recovery coefficient

There are a number of methods to correct for the observed “spill out” of counts from an object due to partial volume effects [16]. The simplest and most widely applied method is to divide the observed count rate by a recovery coefficient determined from phantom data. Dewaraja et al. [19] recommend imaging multiple phantoms of various sizes and geometries using the same acquisition and processing parameters as used for the patient data. An appropriate recovery coefficient is then selected based on the estimated object volume. The recovery coefficient determined from a phantom of volume *v*_true_ is defined as:

32 $$ R=\frac{C}{C_{\mathrm{total}}}, $$

where *C* is the observed count rate measured within a VOI matching the true volume of the phantom and *C*_total_ is the count rate of all counts originating from the phantom [18].

A dataset comprising a series of volumes and the corresponding factors on the right side of Eq. 32 is fitted by an empirical function of appropriate form. Such a function will have adjustable parameters ***b*** = [*b*_1_,…, *b*_*q*_]^T^ and will provide a means for estimating a recovery factor specific to the volume under investigation. The standard uncertainty *u*(*R*) associated with the recovery factor can be derived from the fitted parameters ***b***. A covariance matrix ***V***_***b***_ of dimensions *q* × *q* corresponds to the estimate of ***b*** determined by ordinary least squares fitting under the assumption of equal uncertainty in all data points that make up the dataset. For a perfectly specified volume *v*, the squared standard uncertainty associated with *R* is, using Eq. 8:

33 $$ {u}^2(R)={\boldsymbol{g}}_{\boldsymbol{b}}^{\top }{\boldsymbol{V}}_{\boldsymbol{b}}{\boldsymbol{g}}_{\boldsymbol{b}}, $$

where ***g***_***b***_ is the matrix of dimension *q* × 1 containing the partial derivatives of first order of *R* with respect to ***b***. The covariance matrix ***V***_***b***_ can be determined as a by-product of the least squares fitting process. In reality the standard uncertainty *u*(*R*) obtained in this manner for a given volume will underestimate the actual uncertainty. To provide a more realistic value for *u*(*R*), the standard uncertainty *u*(*v*) associated with the clinical outlined volume *v* has to be taken into consideration. Since this volume is independent of the recovery curve parameters, there will be no covariance associated with *v* and any of the *b*_*j*_. Accordingly, ***V***_***b***_ in Eq. 33 is replaced by:

34 $$ {\boldsymbol{V}}_{\left[\boldsymbol{b},\boldsymbol{v}\right]}=\left[\begin{array}{cc}{\boldsymbol{V}}_{\boldsymbol{b}}& \mathbf{0}\\ {}{\mathbf{0}}^{\top }& {u}^2(v)\end{array}\right], $$

where **0** is a matrix of zeros of dimension *q* × 1 and ***g***_***b***_ is extended by one element, namely the partial derivative of first order of *R*(*v*) with respect to *v*.

### Calibration factor

The final conversion of a partial volume-corrected count rate to activity is achieved by the use of a quantification or calibration factor. The sensitivity of the system is determined by measuring the total count rate *C*_ref_ of a source of known activity *A*_cal_, commonly referred to as a “standard”, under the same acquisition and reconstruction conditions as the study data:

35 $$ Q=\frac{C_{\mathrm{ref}}}{A_{\mathrm{cal}}}. $$

The quantification factor will therefore depend on the standard activity measurement within the dose calibrator and the reconstructed count rate, and its associated uncertainty accordingly calculated. Methods to determine dose calibrator uncertainty are described by Gadd et al. [20]. Uncertainty in the measurement of reconstructed counts within the standard should be determined statistically from a series of nominally identical observations. The uncertainty of a single measurement can be obtained by calculating the mean and standard deviation of that series. The standard uncertainty *u*(*Q*) associated with *Q* can be determined by Eq. 11, a variant of LPU [14], that combines the fractional uncertainties of the dose calibrator measurement and repeated count measurements in quadrature:

36 $$ {\left[\frac{u(Q)}{Q}\right]}^2={\left[\frac{u\left({A}_{\mathrm{cal}}\right)}{A_{\mathrm{cal}}}\right]}^2+{\left[\frac{u\left({C}_{\mathrm{ref}}\right)}{C_{\mathrm{ref}}}\right]}^2. $$

### Activity

The expression relevant to the assessment of the uncertainty associated with the activity *A*_*i*_ determined from the measured count rate *C*_*i*_ in a target VOI at time *t*_*i*_ is:

37 $$ {A}_i=\frac{C_i}{QR}, $$

Equation 37 corresponds to all measurement times *t*_*i*_, for *i* = 1, …, *n*. Using matrix notation:

38 $$ \boldsymbol{A}=\left[\begin{array}{c}{A}_1\\ {}\vdots \\ {}{A}_n\end{array}\right]=\frac{1}{QR}\left[\begin{array}{c}{C}_1\\ {}\vdots \\ {}{C}_n\end{array}\right]. $$

Equation 38 is a multivariate measurement model (section The law of propagation of uncertainty) with *n* + 2 input quantities *Q*, *R*, *C*_1_, …, *C*_*n*_ and *n* output quantities ***A*** = [*A*_1_, …, *A*_*n*_]^⊤^. With no loss of generality, in order to keep the mathematical expressions simpler than they otherwise would be, only two of these activity values, namely, *A*_*i*_ and *A*_*j*_, are considered, measured at times *t*_*i*_ and *t*_*j*_, respectively:

39 $$ \boldsymbol{A}=\left[\begin{array}{c}{A}_i\\ {}{A}_j\end{array}\right]=\frac{1}{QR}\left[\begin{array}{c}{C}_i\\ {}{C}_j\end{array}\right]. $$

Equation 39 is a bivariate measurement model with four input quantities *Q*, *R*, *C*_*i*_ and *C*_*j*_, and two output quantities *A*_*i*_ and *A*_*j*_. Using Eq. 7,

40 $$ {G}_{\left[Q,R,{C}_i,{C}_j\right]}=\left[\begin{array}{cccc}-\frac{A_i}{Q}& -\frac{A_i}{R}& \frac{A_i}{C_i}& 0\\ {}-\frac{A_j}{Q}& -\frac{A_j}{R}& 0& \frac{A_j}{C_j}\end{array}\right], $$

and the input covariance matrix is:

41 $$ {\boldsymbol{V}}_{\left[Q,R,{C}_i,{C}_j\right]}=\left[\begin{array}{cccc}{u}^2(Q)& u\left(Q,R\right)& u\left(Q,{C}_i\right)& u\left(Q,{C}_j\right)\\ {}u\left(Q,R\right)& {u}^2(R)& u\left(R,{C}_i\right)& u\left(R,{C}_j\right)\\ {}u\left(Q,{C}_i\right)& u\left(R,{C}_i\right)& {u}^2\left({C}_i\right)& u\left({C}_i,{C}_j\right)\\ {}u\left(Q,{C}_j\right)& u\left(R,{C}_j\right)& u\left({C}_i,{C}_j\right)& {u}^2\left({C}_j\right)\end{array}\right]. $$

Since *Q* is independent of volume and hence independent of *R*, *C*_*i*_ and *C*_*j*_, the covariance terms *u*(*Q*, *R*), *u*(*Q*, *C*_*i*_) and *u*(*Q*, *C*_*j*_) are zero. Application of Eq. 7 then gives:

42 $$ {\boldsymbol{V}}_{\boldsymbol{A}}={\boldsymbol{G}}_{\left[Q,R,{C}_i,{C}_j\right]}{\boldsymbol{V}}_{\left[Q,R,{C}_i,{C}_j\right]}{\boldsymbol{G}}_{\left[Q,R,{C}_i,{C}_j\right]}^{\top }=\left[\begin{array}{cc}{u}^2\left({A}_i\right)& u\left({A}_i,{A}_j\right)\\ {}u\left({A}_i,{A}_j\right)& {u}^2\left({A}_j\right)\end{array}\right] $$

as the covariance matrix for *A*_*i*_ and *A*_*j*_, the elements of which may be expressed as:

43 $$ {\left[\frac{u\left({A}_i\right)}{A_i}\right]}^2={\left[\frac{u(Q)}{Q}\right]}^2+{\left[\frac{u(R)}{R}\right]}^2+{\left[\frac{u\left({C}_i\right)}{C_i}\right]}^2-2\frac{u\left(R,{C}_i\right)}{RC_i}, $$

44 $$ \frac{u\left({A}_i,{A}_j\right)}{A_i{A}_j}={\left[\frac{u(Q)}{Q}\right]}^2+{\left[\frac{u(R)}{R}\right]}^2+\frac{u\left({C}_i,{C}_j\right)}{C_i{C}_j}-\frac{u\left(R,{C}_i\right)}{RC_i}-\frac{u\left(R,{C}_j\right)}{RC_j}\kern1em \left(I\ne j\right). $$

It follows that the covariances *u*(*R*, *C*_*i*_) and *u*(*C*_*i*_, *C*_*j*_) associated with *Q*, *R* and the *C*_*i*_ must be derived.

Regarding *u*(*C*_*i*_, *C*_*j*_) for this situation, Eq. 4 or, more directly, by the use of formula F.2 in the GUM [14], yields:

45 $$ u\left({C}_i,{C}_j\right)=\frac{\partial {C}_i}{\partial v}\frac{\partial {C}_j}{\partial v}{u}^2(v). $$

Using Eqs. 28 and 30 gives:

46 $$ u\left({C}_i,{C}_j\right)=\frac{\varphi {C}_i}{2 Rv}\frac{{\varphi C}_j}{2 Rv}{u}^2(v). $$

Hence:

47 $$ \frac{u\left({C}_i,{C}_j\right)}{C_i{C}_j}={\left[\frac{\varphi }{2 Rv}\right]}^2{u}^2(v)={\left[\frac{u\left({C}_i\right)}{C_i}\right]}^2. $$

As both the recovery coefficient *R* and the measured count rate *C*_*i*_ depend on the VOI outline they can be expressed as functions of volume *v*. Again applying the GUM [14] (formula F.2) and using Eqs. 28 and 30 gives:

48 $$ u\left(R,{C}_i\right)=\frac{\varphi {C}_i}{2 Rv}\frac{\partial R}{\partial v}{u}^2(v), $$

which, after rearrangement, can be expressed as:

49 $$ \frac{u\left(R,{C}_i\right)\ }{R{C}_i}=\frac{u\left(R,{C}_j\right)\ }{R{C}_j}=\frac{\varphi }{2{R}^2v}\frac{\partial R}{\partial v}{u}^2(v). $$

After substituting the covariance expressions of Eqs. 47 and 49 into Eqs. 43 and 44 it can be seen that:

50 $$ {\left[\frac{u\left({A}_i\right)}{A_i}\right]}^2=\frac{u\left({A}_i,{A}_j\right)}{A_i{A}_j}={\left[\frac{u(Q)}{Q}\right]}^2+{\left[\frac{u(R)}{R}\right]}^2+{\left[\frac{u\left({C}_i\right)}{C_i}\right]}^2-\frac{\varphi }{R^2v}\frac{\partial R}{\partial v}{u}^2(v) $$

Given the equal fractional uncertainties for all the *A*_*i*_ and with perfect covariance between the *A*_*i*_ and *A*_*j*_, it is appropriate to treat these uncertainties in a manner similar to a systematic error. Hence the fractional uncertainties in activity can be propagated into a systematic component of uncertainty for cumulated activity${u}_s\left(\overset{\sim }{A}\right)$, where

51 $$ {\left[\frac{u\left({A}_i\right)}{A_i}\right]}^2={\left[\frac{u_s\left(\overset{\sim }{A}\right)}{\overset{\sim }{A}}\right]}^2. $$

### Time–activity curve parameters

In addition to the systematic uncertainties associated with quantification and volume determination, uncertainties in the TAC data can arise from other sources such as image noise, patient motion, registration and other imperfect post-acquisition operations such as image reconstruction, including scatter and attenuation corrections. Due to the complexity of these operations, it is assumed that the uncertainties associated with the compensation for effects such as attenuation and scatter are negligible in comparison with the uncertainty associated with the compensation for partial volume effects [13]. It is therefore more appropriate to measure the causality of imperfects in these corrections, and to derive uncertainties in the fit parameters of the TAC.

Estimates of the TAC parameters ***p*** = [*p*_1_,…, *p*_*q*_]^⊤^ can be determined by fitting data points (*t*_*i*_, *A*_*i*_),  *i* = 1, …, *n*, where the *t*_*i*_ denote the image acquisition times and *A*_*i*_ the corresponding measured activities. A least squares approach is recommended, using nonlinear regression techniques to minimize the objective function

52 $$ {\chi}^2=\sum {\left[{A}_i-f\left({t}_i\right)\right]}^2 $$

with respect to ***p***. Note that a weighting term to account for the activity uncertainty is not included due to the covariant nature of the uncertainty. Uncertainties of the fit parameters are then estimated using:

53 $$ {\boldsymbol{V}}_{\boldsymbol{p}}=\frac{{\boldsymbol{\chi}}^{\mathbf{2}}}{n-q}{\left[{\boldsymbol{J}}_{\boldsymbol{p}}^{\top }{\boldsymbol{J}}_{\boldsymbol{p}}\right]}^{-1} $$

where ***J***_***p***_ is the matrix of first-order partial derivatives of the TAC model with respect to ***p***, evaluated at ***A***. The TAC is generally represented as a sum of exponential functions. For the purpose of presentation, only the case of a single exponential function is described:

54 $$ f(t)=A(t)={A}_0{e}^{-\lambda t}, $$

where *A*_0_ is the activity at time zero and *λ* is the effective decay constant, for which

55 $$ {\boldsymbol{J}}_{\boldsymbol{p}}=\left[\begin{array}{cc}\frac{\partial {A}_1}{\partial {A}_0}& \frac{\partial {A}_1}{\mathrm{\partial \uplambda }}\\ {}\vdots & \vdots \\ {}\frac{\partial {A}_n}{\partial {A}_0}& \frac{\partial {A}_n}{\mathrm{\partial \uplambda }}\end{array}\right]=\left[\begin{array}{cc}{e}^{-\lambda {t}_1}& -{A}_0{t}_1{e}^{-\lambda {t}_1}\\ {}\vdots & \vdots \\ {}{e}^{-\lambda {t}_n}& -{A}_0{t}_n{e}^{-\lambda {t}_n}\end{array}\right] $$

and

56 $$ {\boldsymbol{V}}_{\boldsymbol{p}}=\left[\begin{array}{cc}{u}^2\left({A}_0\right)& u\left({A}_0,\lambda \right)\\ {}u\left({A}_0,\lambda \right)& {u}^2\left(\lambda \right)\end{array}\right]. $$

### Cumulated activity

The cumulated activity is defined as the integral of the TAC from time *t* = 0 to ∞, which for a single exponential function is described simply by the ratio of the TAC parameters, that is:

57 $$ \overset{\sim }{A}={\int}_0^{\infty }A(t)\mathrm{d}t={\int}_0^{\infty }{A}_0{e}^{-\lambda t}\ \mathrm{d}t=\frac{A_0}{\lambda }. $$

Application of Eqs. 8 and 9 to Eq. 57 is used to derive the component of uncertainty associated with random effects:

58 $$ {\mathrm{u}}_r^2\left(\overset{\sim }{A}\right)={\boldsymbol{g}}_{\boldsymbol{p}}^{\top }{\boldsymbol{V}}_{\boldsymbol{p}}{\boldsymbol{g}}_{\boldsymbol{p}} $$

where

59 $$ {\boldsymbol{g}}_{\boldsymbol{p}}^{\top }=\left[\frac{\partial \overset{\sim }{A}}{\partial {A}_0},\kern0.5em \frac{\partial \overset{\sim }{A}}{\mathrm{\partial \uplambda }}\right]=\left[\frac{1}{\lambda },\kern0.5em -\frac{A_0}{\lambda^2}\right], $$

and ***V***_***p***_ is the covariance matrix for the estimates of the TAC parameters ***p =*** [*A*_0_, *λ*]^⊤^given in Eq. 53. After expansion of these matrices the component of uncertainty associated with random effects in $\overset{\sim }{A}$ can be expressed as:

60 $$ {\left[\frac{u_r\left(\overset{\sim }{A}\right)}{\overset{\sim }{A}}\right]}^2={\left[\frac{u\left({A}_0\right)}{A_0}\right]}^2+{\left[\frac{u\left(\lambda \right)}{\lambda}\right]}^2-2\frac{u\left({A}_0,\lambda \right)\ }{A_0\lambda }. $$

Random and systematic components can be combined by considering the general model:

61 $$ x={x}^{\mathrm{nom}}+r+s, $$

where *x*^nom^ is the nominal value of some parameter *x*, and *r* and *s* are random and systematic effects, respectively. Then, applying LPU:

62 $$ {u}^2(x)={u}^2(r)+{u}^2(s). $$

For cumulated activity

63 $$ {u}^2\left(\overset{\sim }{A}\right)={u}_r^2\left(\overset{\sim }{A}\right)+{u}_s^2\left(\overset{\sim }{A}\right), $$

hence, using Eqs. 50, 51 and 60:

64 $$ {\left[\frac{u\left(\overset{\sim }{A}\right)}{\overset{\sim }{A}}\right]}^2={\left[\frac{u\left({A}_0\right)}{A_0}\right]}^2+{\left[\frac{u\left(\lambda \right)}{\lambda}\right]}^2-2\frac{u\left({A}_0,\lambda \right)\ }{A_0\lambda }+{\left[\frac{u(Q)}{Q}\right]}^2+{\left[\frac{u(R)}{R}\right]}^2+{\left[\frac{u\left({C}_I\right)}{C_I}\right]}^2-\frac{\varphi }{R^2v}\frac{\partial R}{\partial v}{u}^2(v). $$

### S-factor

**Fig. 6:**
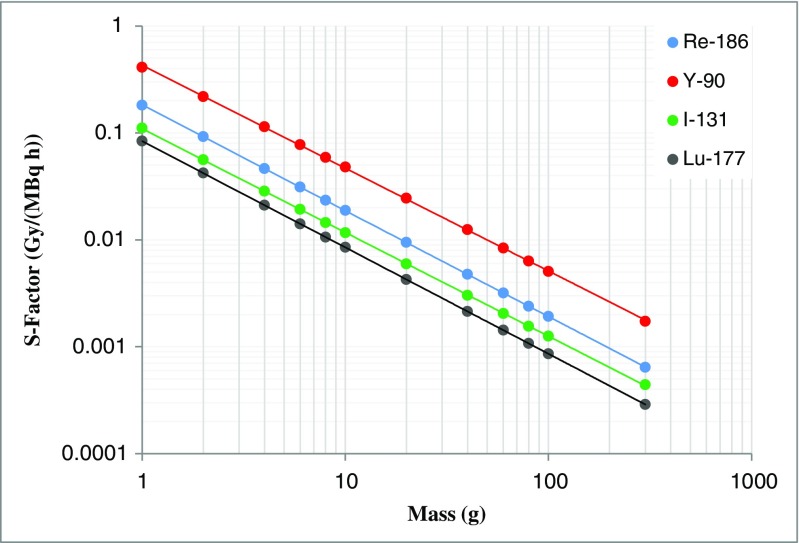
Example plot of S-factor versus mass for the radionuclides indicated for unit density spheres

Uncertainties associated with S-factors are somewhat less complicated than in the case of cumulated activity, and are predominantly influenced by the uncertainty associated with the volume. The general approach to determining S-factors is to choose a value calculated for a model that closely approximates the organ or region of interest. If a model of the corresponding size does not exist, a scaling can be applied to adjust the S-factor accordingly. Alternatively, an empirical S-factor versus mass representation can be obtained by fitting suitable S-factor data against mass [21, 22]. The implicit assumption is that appropriate models exist for the clinical situation. There are uncertainties associated with deviations between the model and reality (for example, a tumour that is not spherical) but these are outside the scope of this framework. Figure 6 shows, on a log-log scale, an example of S-factor data for unit density spheres of different masses [23], empirically fitted by the function:

65 $$ S={c}_1{m}^{-{c}_2}. $$

It is possible to apply the same principles as employed in the previous section to determine a covariance matrix for the estimated parameters in the fitting function. However, in this instance the standard uncertainties associated with these fit parameters tend to be very small (<1%) and the mass uncertainty dominates. Therefore, these estimated parameter uncertainties can be ignored and, using Eq. 31, the standard uncertainty in *S* is:

66 $$ u(S)=\left|\frac{\partial S}{\partial m}\right|u(m)=\left|-{c}_1{c}_2{m}^{-{c}_2-1}\right|u(m)=\left|{c}_2\right|\frac{S}{m}u(m). $$

Given that mass is proportional to volume, and assuming a known tissue density with negligible uncertainty, the fractional standard uncertainty associated with *S* is:

67 $$ \frac{u(S)}{S}=\left|{c}_2\right|\frac{u(v)}{v}. $$

The fractional standard uncertainty associated with *S* is thus proportional to the fractional standard uncertainty associated with volume *v*, the proportionality constant being the magnitude |*c*_2_| of the slope of the fitting function on a log-log scale.

### Absorbed dose

Having established standard uncertainty expressions for $\overset{\sim }{A}$ and *S*, it is evident that both parameters have a dependence on volume. To determine a final uncertainty in the absorbed dose, the covariance between these parameters should therefore be determined. Applying the GUM, [15] (formula F.2) the covariance $u\left(\overset{\sim }{A},S\right)$ is evaluated using:

68 $$ u\left(\overset{\sim }{A},S\right)=\frac{\partial \overset{\sim }{A}}{\partial v}\frac{\partial S}{\partial v}{u}^2(v). $$

An expression for$\overset{\sim }{A}$ with respect to volume is difficult to derive. However, using LPU [15] (formula 12) and with only the systematic uncertainty component in $\overset{\sim }{A}$ having a volume dependence,

69 $$ {u}_s\left(\overset{\sim }{A}\right)=\frac{\partial \overset{\sim }{A}}{\partial v}u(v). $$

Using Eq. 51, the fractional standard uncertainty in $\overset{\sim }{A}$ can be replaced with that of activity to give:

70 $$ \frac{\partial {A}_i}{\partial v}\frac{u(v)}{A_i}=\frac{\partial \overset{\sim }{A}}{\partial v}\frac{u(v)}{\overset{\sim }{A}}. $$

Hence,

71 $$ u\left(\overset{\sim }{A},S\right)=\frac{\partial {A}_i}{\partial v}\frac{\partial S}{\partial v}{u}^2(v)\frac{\overset{\sim }{A}}{A_i}. $$

$\frac{\partial S}{\partial v}$ is obtained from Eq. 65: with *v* in place of *m*,

72 $$ \frac{\partial S}{\partial v}=-\frac{c_2}{v}S. $$

To provide  $\frac{\partial {A}_i}{\partial v}$ requires a re-expression of activity as a function of volume. Use of Eq. 37 and differentiating with respect to *v* yields:

73 $$ \frac{\partial {A}_i}{\partial v}=\frac{1}{QR}\frac{\partial {C}_i}{\partial v}-\frac{C_i}{Q{R}^2}\frac{\partial R}{\partial v}. $$

Using Eq. 26 gives:

74 $$ \frac{\partial {A}_i}{\partial v}=\frac{A_i}{R}\ \left(\frac{\varphi }{2v}-\frac{\partial R}{\partial v}\right) $$

such that covariance between$\overset{\sim }{A}$ and*S* can be expressed as:

75 $$ u\left(\overset{\sim }{A},S\right)=-\frac{c_2}{Rv}\overset{\sim }{A}S\ \left(\frac{\varphi }{2v}-\frac{\partial R}{\partial v}\right)\ {u}^2(v). $$

Having established expressions for covariance uncertainty in $\overset{\sim }{A}$ and *S*, Eqs. 75, 67 and 64 can be used in Eq. 8 to give a final uncertainty in absorbed dose, given by:

76 $$ {u}^2\left(\overline{D}\right)={\boldsymbol{g}}_{\left[\overset{\sim }{A,}S\right]}^{\top }{\boldsymbol{V}}_{\left[\overset{\sim }{A},S\right]}{\boldsymbol{g}}_{\left[\overset{\sim }{A},S\right]}, $$

where ${\boldsymbol{g}}_{\left[\overset{\sim }{A},S\right]}$ and ${\boldsymbol{V}}_{\left[\overset{\sim }{A},S\right]}$are the respective gradient and covariance matrices associated with $\overset{\sim }{A}$ and *S* which, using Eq. 13 for the case of a single exponential TAC, can be written:

77 $$ {\displaystyle \begin{array}{c}{\left[\frac{u\left(\overline{D}\right)}{\overline{D}}\right]}^2={\left[\frac{u\left({A}_0\right)}{A_0}\right]}^2+{\left[\frac{u^2\left(\lambda \right)}{\lambda}\right]}^2-2\frac{u\left({A}_0,\lambda \right)\ }{A_0\lambda}\\ {}+{\left[\frac{u(Q)}{Q}\right]}^2+{\left[\frac{u(R)}{R}\right]}^2+{\left[\frac{u\left({C}_i\right)}{C_i}\right]}^2-\frac{\varphi }{R^2v}\frac{\partial R}{\partial v}{u}^2(v)\\ {}+{\left|{c}_2\right|}^2{\left[\frac{u(v)}{v}\right]}^2\\ {}-2\frac{c_2}{Rv}\left(\frac{\varphi }{2v}-\frac{\partial R}{\partial v}\right)\ {u}^2(v).\end{array}} $$

## Patient example

An example to demonstrate the implementation of the approach described in this paper is given in the following sections, with details of the methodology used to obtain the absorbed dose data and the associated uncertainty analysis. The example given is that of a 47-year-old patient who presented with weight loss, lethargy and upper abdominal cramps. Upper gastrointestinal endoscopy showed a mass in the third part of the duodenum. A subsequent contrast-enhanced CT scan and ^68^Ga-DOTATATE PET/CT investigation showed a 6.5-cm mass arising from the pancreatic head and a 3-cm mass within segment 4 of the liver, in keeping with a neuroendocrine tumour arising from the pancreas. The patient underwent ^90^Y-DOTATATE radiopeptide therapy in combination with ^111^In-DOTATATE for imaging. The administered activity was 4,318 MBq of ^90^Y with ^111^In given at a ratio of 1:25.

### Image acquisition

**Fig. 7:**
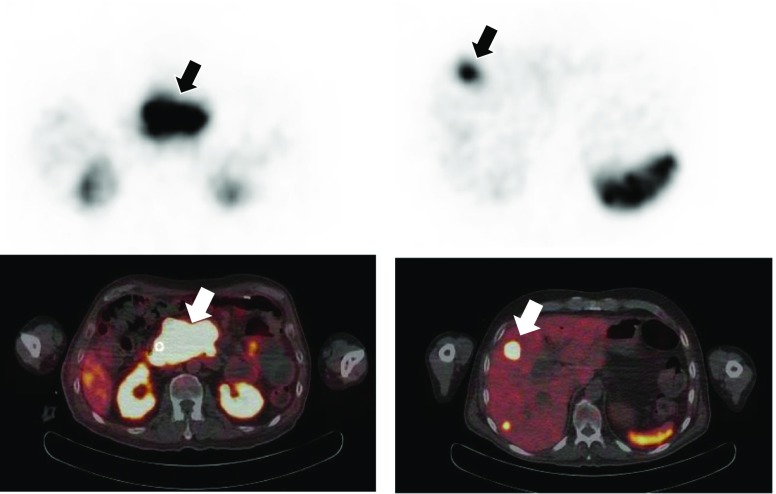
^111^In-DOTATATE SPECT and ^68^Ga-DOTATATE PET/CT images of neuroendocrine tumours in a patient treated with ^90^Y-DOTATATE radiopeptide therapy. *Arrows* indicate the lesions for which doses are to be calculated

Absorbed doses for the lesions were calculated using sequential ^111^In SPECT acquisitions, performed at 19.7 h, 45.1 h and 66.5 h after administration, acquiring 64 projections in a 128 matrix for 60 s per view. Triple-energy window scatter corrections were applied to the projection data with 20% energy windows centred on the 171 keV and 245 keV photopeaks. The scatter-corrected data for each energy window were then added and reconstructed iteratively with a weighted attenuation coefficient based on the photopeak abundance as described by Seo et al. [24]. The reconstructed SPECT voxel size was 4.67 mm. ^111^In-DOTATATE SPECT images are shown in Fig. 7 alongside the ^68^Ga-DOTATATE PET/CT images. The primary tumour and the hepatic lesion are indicated on the images from the two modalities.

### Volume

VOIs of the two lesions were determined using an adaptive thresholding technique, whereby a threshold for outlining was chosen based on the known threshold required to outline similar sized volumes on phantom data. As the VOI was drawn directly on the SPECT data, the voxelization uncertainty was combined with the spatial resolution element, given in Eq. 19. The reconstructed system spatial resolution was determined directly from physical measurements of a point source in air. The measured FWHM was 0.9 cm. Volumes and uncertainty components are shown in Table 1.

**Table 1:** Volumes and associated standard uncertainties for liver and pancreatic lesions

	Volume (cm^3^)	Fractional standard uncertainty (%)		
		Due to voxelization	Due to resolution	Combined
Liver lesion	13.9	19.1	54.4	57.6
Pancreatic lesion	142.0	8.8	25.1	26.6

### Recovery coefficient

**Fig. 8:**
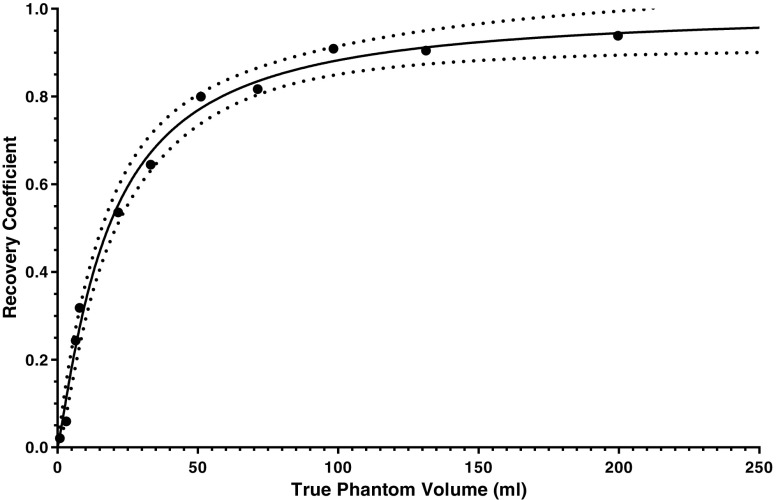
A recovery curve used to correct for partial volume losses for objects of different sizes. The *solid line* indicates the fitted function and the *dotted lines* indicate the lower and upper limits of the 95% confidence interval of the fitted function

Recovery data were generated by imaging multiple phantoms with the same acquisition and processing parameters as described for the patient data. The phantoms consisted of a 20 cm × 12 cm cylindrical phantom within which smaller inserts could be placed. Insert volumes ranged from 0.1 ml to 200 ml and were filled with a known concentration of ^111^In. For each insert two VOIs were drawn. The first set of VOIs were generated by selecting the appropriate percentage threshold to match the known physical volume of the insert. The second set of VOIs were drawn to encompass all counts (including spill out) that originated from the insert volume. A recovery coefficient for each insert was then determined using Eq. 32. The generated recovery curve is given in Fig. 8. The empirical function fitted to the example data takes the form of a two-parameter logistic function, with respect to volume *v* [25], namely:

78 $$ R(v)=1-\frac{1}{1+{\left(\frac{v}{b_1}\right)}^{b_2}}. $$

Fit parameters of the curve with associated uncertainties were determined using GraphPad Prism fitting software (La Jolla, CA, USA) and are detailed in Table 2. The covariance between the parameters was calculated as 0.0155, which can be expressed as a correlation coefficient, *r*, defined as:

79 $$ r\left({b}_1,{b}_2\right)=\frac{u\left({b}_1,{b}_2\right)}{u\left({b}_1\right)u\left({b}_2\right)}=0.213 $$

**Table 2:** Recovery curve fit parameters and associated standard uncertainties

Parameter	Value	Standard uncertainty	Fractional standard uncertainty (%)
*b*_1_	21.1 ml	1.2 ml	5.8
*b*_2_	1.06	0.06	5.6

Equations 33 and 34 were used to combine the volume uncertainties with the recovery coefficient uncertainty for both lesions as shown in Table 3. Uncertainty estimates are given with and without the volume component. The importance of propagating the volume uncertainty into the calculation is clearly apparent for the smaller of the two lesions.

**Table 3:** Recovery coefficients and associated standard uncertainties with and without the volume component for liver and pancreatic lesions

	*R*	*u*(*R*)_[*b*]_/*R*	*u*(*R*)_[*b*,*v*]_/*R*
Liver lesion	0.39	4.3%	37.4%
Pancreatic lesion	0.88	1.4%	3.6%

### Count rate

Count rates for each lesion with each scan time are shown in Table 4 with the associated fractional standard uncertainties. The uncertainty in the VOI count rate is described in Eq. 31.

**Table 4:** Count rates and associated standard uncertainties for liver and pancreatic lesions

VOI	Count rate (cps)			Fractional standard uncertainty (%)
	Scan 1	Scan 2	Scan 3	
Liver lesion	56.8	23.2	18.0	58.6
Pancreatic lesion	865	480	292	13.6

The covariance between the recovery coefficient and count rates, *u*(*R*,*C*), is defined in Eq. 48, which when combined with the empirical function given in Eq. 78 can be re-expressed as:

80 $$ u\left({C}_I,R\right)=\frac{\varphi {C}_I{b}_2{\left(\frac{v}{b_1}\right)}^{b_2}}{2R{v}^2{\left(1+{\left(\frac{v}{b_1}\right)}^{b_2}\right)}^2}{u}^2(v). $$

Substitution of the fit parameters, volumes and count rates into this expression is used to generate the values for covariance which are shown in Table 5. Correlation coefficients relating to these covariance values are 0.99 and 0.92 for the liver and pancreatic lesion, respectively.

**Table 5:** Covariance values of count rate and recovery coefficient at each scan for the liver and pancreatic lesions

Scan	*u*(*R*,*C*)	
	Liver lesion	Pancreatic lesion
1	4.84	3.42
2	1.98	1.90
3	1.54	1.16

### Calibration

**Fig. 9:**
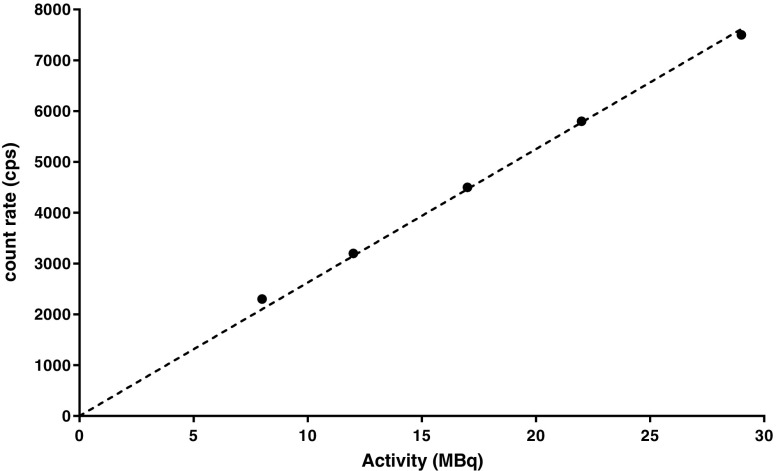
Activity versus count rate of reconstructed point sources in air

The system was calibrated by imaging point sources of ^111^In at various activities (8 MBq to 30 MBq) in air using the same acquisition parameters as for the patient scans. Images were reconstructed according to the clinical protocol and a spherical VOI was placed over the reconstructed point, ensuring that all counts from the source were contained. A plot of VOI count rate versus activity is given in Fig. 9.

The fractional standard uncertainty in activity was taken as 1.5%, the typical uncertainty for secondary standard calibrators for ^111^In as given by Gadd et al. [20]. The statistical uncertainty from repeating the calibration measurement was taken from the standard deviation of the mean. Combining these uncertainties, as shown in Eq. 36, yields:

$$ \mathrm{Q}=275\ \mathrm{cps}/\mathrm{MBq},\mathrm{with}\ \mathrm{a}\ \mathrm{standard}\ \mathrm{error}\ \mathrm{of}\ 8\ \mathrm{cps}/\mathrm{MBq}. $$

### Activity

Calculation of ^111^In activity within each lesion is obtained from Eq. 37 using the measured count rate *C*_*i*_, volume-specific recovery coefficient *R* and calibration factor *Q*. ^111^In activity was converted to ^90^Y activity by scaling the ratio of the administered activities and correcting for decay according to the different half-lives of the two isotopes, such that the ^90^Y activity is expressed as:

81 $$ A{\left({}^{90}\mathrm{Y}\right)}_t=\frac{A{\left({}^{90}\mathrm{Y}\right)}_{\mathrm{admin}}A\Big({{}^{111}\mathrm{In}\Big)}_t}{A{\left({}^{111}\mathrm{In}\right)}_{\mathrm{admin}}}\times {e}^{\left({\lambda}_{111\mathrm{In}}-{\lambda}_{90\mathrm{Y}}\right)t} $$

The variance associated with the measured activity is given in Eq. 50 and assumes negligible uncertainty in the administered isotope activities. It is therefore a simple case of substituting the relevant variance and covariance values for *Q*, *R* and *C* to form the required uncertainty in *A*_*i*_. These activities and associated uncertainties are shown in Table 6.

**Table 6:** ^90^Y activities and associated fractional standard uncertainty for liver and pancreatic lesions

VOI	^90^Y activity (MBq)			Fractional standard uncertainty (%)
	Scan 1	Scan 2	Scan 3	
Liver lesion	13.1	5.3	4.0	22.1
Pancreatic lesion	88.3	48.2	29.0	10.9

### TAC fitting

**Fig. 10:**
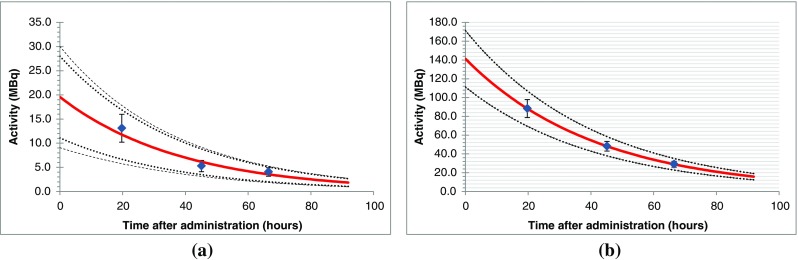
TAC for liver (**a**) and pancreatic (**b**) lesions. *Error bars* for each point are the standard uncertainty of the measured activity. *Dotted lines* indicate the 95% confidence intervals due to systematic uncertainty in activity combined with the parameter uncertainties in the fitting algorithm

The Gauss-Newton algorithm was used to minimize the objective function described by Eq. 52. A single exponential function was fitted to the data and the uncertainties in the fit parameters *A*_0_ and *λ* were determined using Eq. 53. TACs for the two lesions are given in Fig. 10 with the fitted exponential functions. Error bars on the data points represent the estimated standard uncertainty in activity.

Solution parameters with associated random and systematic components of uncertainty for each TAC are shown in Table 7.

**Table 7:** TAC parameters and associated standard uncertainties for liver and pancreatic lesions

	Liver lesion			Pancreatic lesion		
	Fitted value	Standard uncertainty	Fractional uncertainty (%)	Fitted value	Standard uncertainty	Fractional uncertainty (%)
*A*_0_ (fitting) (MBq)	19.6	5.10	26.1	141.2	0.2	0.1
*λ* (fitting) (h^−1^)	0.026	0.008	32.4	0.0238	3.0 × 10^−5^	0.1

### Cumulated activity

The covariance matrix for the solution parameters ***V***_***p***_ is given in Table 8. The product of the covariance matrix and the gradient matrix ***g***_***p***_ was used to determine the random component of the variance of $\overset{\sim }{A}$ described in Eq. 58.

**Table 8:** Covariance and gradient matrices used to calculate random (fitting) component of uncertainty in cumulated activity for liver and pancreatic lesions

	Covariance matrix ***V***_***p***_	Gradient matrix ***g***_***p***_	Fractional standard uncertainty in $\overset{\sim }{A}$ (%)
Liver lesion	$$ \left[\begin{array}{cc}26.1& 3.77\times {10}^{-2}\\ {}3.77\times {10}^{-2}& 6.91\times {10}^{-5}\end{array}\right] $$	$$ \left[\begin{array}{c}39.00\\ {}-29722\end{array}\right] $$	15.1
Pancreatic lesion	$$ \left[\begin{array}{cc}0.0240& 4.66\times {10}^{-6}\\ {}4.66\times {10}^{-6}& 1.15\times {10}^{-9}\end{array}\right] $$	$$ \left[\begin{array}{c}42.0\\ {}-2.49\times {10}^5\end{array}\right] $$	0.1

Random and systematic components of uncertainty in the cumulated activity were determined according to Eqs. 50 and 60. These results and that of combined uncertainty (Eq. 63) are shown in Table 9.

**Table 9:** Cumulated activity and associated components of standard uncertainties for liver and pancreatic lesions

	Liver lesion			Pancreatic lesion		
	Value (MBq h)	$\mathrm{u}\left(\overset{\sim }{\mathrm{A}}\right)$ (MBq h)	Fractional uncertainty (%)	Value (MBq h)	$\mathrm{u}\left(\overset{\sim }{\mathrm{A}}\right)$ (MBq h)	Fractional uncertainty (%)
$\overset{\sim }{A}$ (fitting)		115	15.1		4.0	0.0678
$\overset{\sim }{A}$ (systematic)	762	168	22.1	5,933	644	10.9
$\overset{\sim }{A}$ (total)		204	26.7		644	10.9

### S-factors

The S-factors for the lesions were determined by fitting ^90^Y S-factor data for unit density spheres against mass [21], empirically fitted by the function:

82 $$ S={c}_1{m}^{-{c}_2}. $$

The fit parameters of the curve with associated uncertainty were determined using GraphPad fitting software (La Jolla, CA, USA) and are shown in Table 10. As the standard uncertainties associated with these fit parameters are much less than the mass uncertainty of the two lesions the estimated parameter uncertainties can be ignored and Eq. 66 holds. Table 11 shows the determined S-factors for the lesions with associated uncertainties.

**Table 10:** Fit parameters and associated standard uncertainties for S-factor data of unit density spheres

Parameter	Fitted value	Standard uncertainty	Fractional uncertainty (%)
*c*_1_	0.429	3.7 × 10^−3^	0.4
*c*_2_	−0.961	5.1 × 10^−3^	1.2

**Table 11:** Summary of VOI S-factor data with standard uncertainties *u*(*S*)

	S-factor (Gy/MBq h)	Standard uncertainty	Fractional uncertainty (%)
Liver lesion	3.4 × 10^−2^	1.9 × 10^−2^	55.5
Pancreatic lesion	3.7 × 10^−3^	0.9 × 10^−3^	25.5

### Absorbed dose

The uncertainty in the absorbed dose is determined from Eq. 13, for which the covariance $u\left(\overset{\sim }{A},S\right)$ is required. Use of Eq. 75 to determine this covariance requires solving the partial derivative $\frac{\partial R}{\partial v}$ and substituting the determined parameters $S,\overset{\sim }{A},R,v,{c}_1,{c}_2$ and the standard uncertainty *u*(*v*). For the recovery function defined in Eq. 78, the partial derivative is expressed as:

83 $$ \frac{\partial R}{\partial v}=\frac{b_2{\left(\frac{v}{b_1}\right)}^{b_2}}{v{\left(1+{\left(\frac{v}{b_1}\right)}^{b_2}\right)}^2} $$

Solutions for $u\left(\overset{\sim }{A},S\right)$ are shown in Table 12. It can be seen from the correlation coefficients that the covariance between $\overset{\sim }{A}$ and *S* is highly significant. In addition, the negative nature of the correlation results in a reduction in the final absorbed dose uncertainty, as shown in Table 13.

**Table 12:** VOI S-factor data with standard uncertainties *u*(*S*)

	$\overset{\sim }{\mathrm{A}}$	$\mathrm{u}\left(\overset{\sim }{\mathrm{A}}\right)$	S (Gy/MBq h)	*u*(*S*) (Gy/MBq h)	$\mathrm{u}\left(\overset{\sim }{\mathrm{A}},\mathrm{S}\right)$	$r\left(\overset{\sim }{\mathrm{A}},\mathrm{S}\right)$
Liver lesion	762	203	3.4 × 10^−2^	1.9 × 10^−2^	−3.09	−0.80
Pancreatic lesion	5,932	1,046	3.7 × 10^−3^	0.9 × 10^−3^	−0.57	−0.95

**Table 13:** Absorbed dose parameters and associated standard uncertainties for liver and pancreatic lesions

	Absorbed dose (Gy)	Covariance matrix, ${V}_{\left[\overset{\sim }{A},S\right]}$	Gradient matrix, ${g}_{\left[\overset{\sim }{A},S\right]}$	Fraction uncertainty in $\overline{D}$ (%)
Liver lesion	26.1	$$ \left[\begin{array}{cc}4.16\times {10}^4& -3.09\\ {}-3.09& 3.60\times {10}^{-4}\end{array}\right] $$	$$ \left[\begin{array}{c}0.0342\\ {}762\end{array}\right] $$	37.6
Pancreatic lesion	21.7	$$ \left[\begin{array}{cc}4.15\times {10}^5& -0.572\\ {}-0.572& 8.71\times {10}^{-7}\end{array}\right] $$	$$ \left[\begin{array}{c}0.00365\\ {}5932\end{array}\right] $$	15.6

**Fig. 11:**
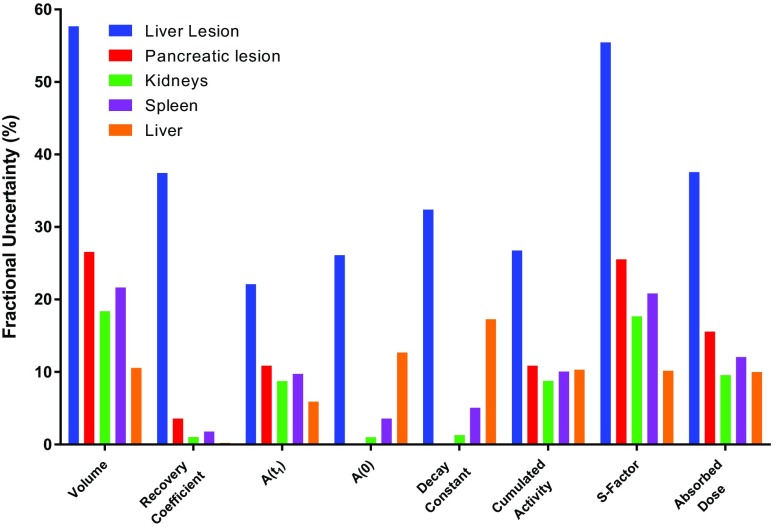
Fractional uncertainty of calculated dosimetric parameters for lesions and normal organs

Propagation of uncertainty can be visualized by examination of the fractional uncertainty of each parameter calculated along the dosimetry chain. Figure 11 gives uncertainties for the absorbed doses delivered to lesions and to normal organs. It can be seen that the small volume of the liver lesion has a significant impact on the larger fractional uncertainty compared to the larger lesion and organ volumes.

**Fig. 12:**
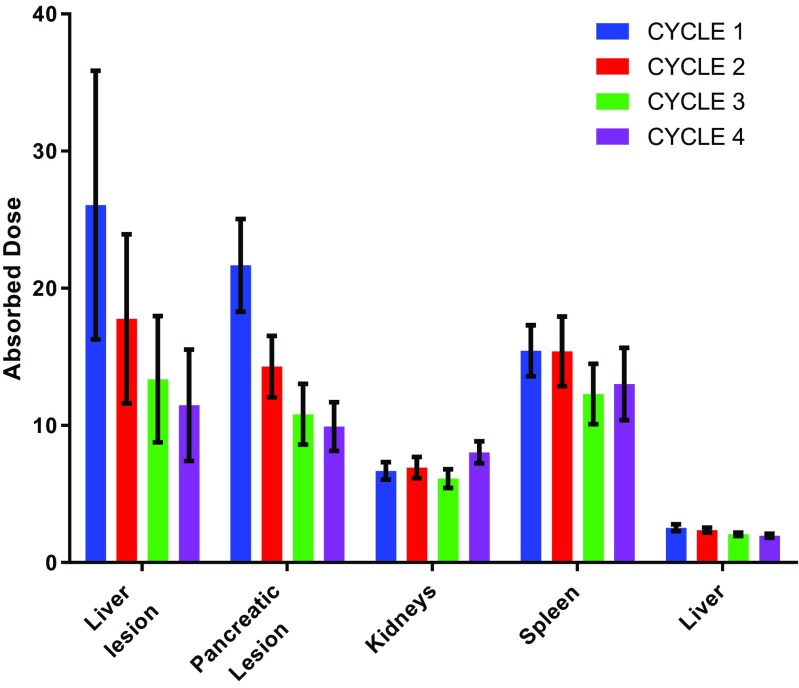
Absorbed doses to lesions and normal organs over four treatment cycles. *Error bars* represent standard uncertainties in the dose values

Using the methodology described, absorbed doses to lesions and normal organs were calculated. In addition, the treatment was repeated for four cycles and an equivalent methodology was employed. Dosimetry results for lesions and normal organs delivered at each fraction are presented in Table 14 and shown graphically in Fig. 12. A significant decrease in absorbed dose to the lesions was observed after the first cycle and an increase in absorbed dose to the kidneys after the fourth cycle.

**Table 14:** Absorbed doses with standard uncertainties for lesions and normal organs over treatment cycles

	Cycle 1		Cycle 2		Cycle 3		Cycle 4	
	$\overline{D}$ (Gy)	$u\left(\overline{D}\right)$ (Gy)	$\overline{D}$ (Gy)	$u\left(\overline{D}\right)$ (Gy)	$\overline{D}$ (Gy)	$u\left(\overline{D}\right)$ (Gy)	$\overline{D}$ (Gy)	$u\left(\overline{D}\right)$ (Gy)
Liver lesion	26.1	9.8	17.8	6.2	13.4	4.6	11.5	4.1
Pancreatic lesion	21.7	3.4	14.3	2.2	10.8	2.2	9.9	1.8
Kidneys	6.68	0.3	6.9	0.8	6.1	0.7	8.0	0.8
Spleen	15.4	1.9	15.4	2.5	12.3	2.2	13.0	2.6
Liver	2.54	0.6	2.4	0.2	2.1	0.1	2.0	0.2

### Propagation of uncertainties from a tracer to a therapy study

A further potential source of uncertainty is the use of a pretherapy or concomitant diagnostic tracer study to predict the absorbed dose that would be delivered from a different therapeutic agent. It has previously been shown that uncertainty in the estimation of the biological half-life of the tracer will have an impact on the uncertainty of the absorbed dose calculated for the therapy procedure as a function of the relative values of the biological and physical half-lives [6]. The uncertainty in the therapeutic effective half-life can then be expressed as;

$$ u\left({T}_{\mathrm{eff}\ \left(\mathrm{th}\right)}\right)=u\left({T}_{\mathrm{eff}\ \left(\mathrm{tr}\right)}\right)\frac{T_{\mathrm{eff}\ \left(\mathrm{th}\right)}}{T_{\mathrm{eff}\ \left(\mathrm{tr}\right)}} $$

where *T*_ eff (th)_ and *T*_eff (tr)_ are the effective half-lives of the therapeutic and the diagnostic radionuclide, respectively.

In the limiting case of infinite biological retention, the ratio of the uncertainties for the tracer and therapeutic agent will be the ratio of the physical half-lives. An uncertainty in the absorbed dose calculated for ^111^In (physical half-life *T*_phys_ = 67.3 h) will therefore produce a similar uncertainty in the absorbed dose for a ^90^Y calculation (*T*_phys_ = 64.1 h). However, a small uncertainty in, for example, an absorbed dose calculation for ^68^Ga (*T*_phys_ = 1.13 h) would propagate by a factor of ~60 to give potentially significant uncertainty in a ^90^Y calculation.

It is important to note that nonconformance, for example different administered amounts or affinities between diagnostic and therapeutic radiopharmaceuticals, will also introduce additional uncertainties into the prediction [26, 27]. For example, it is assumed that the biokinetics of ^111^In- and ^90^Y-DOTATATE are equal, whereas the renal uptake of the indium-labelled compound might be higher [28].

## Discussion

The methodology presented allows uncertainty analysis to be incorporated into absorbed dose calculations using the MIRD schema [1], the most widely adopted approach for molecular radiotherapy (MRT) dosimetry. The methodology is based on the recommendations described within the GUM [14] and necessarily involves the formation of covariance matrices for several steps of the dosimetry process.

The main objective of this uncertainty propagation schema is to evaluate the standard uncertainty in absorbed dose to a target. The tasks that directly support that objective are the determination of cumulated activity and the S-factor. The cumulated activity, given by the area under a TAC, is obtained from a sequence of quantitative images. Each activity value is expressed in terms of an observed count rate, a calibration factor and a recovery coefficient. The recovery coefficient is based on a recovery curve derived from multiple phantom scans. The presence of a common calibration and recovery factor in all activity values, and the covariance between volume, recovery and measured count rate, can be considered as a systematic uncertainty applied across all TAC data points, and therefore may be applied directly to cumulated activity. For the effects of uncertainties associated with random components of TAC data, a statistical approach using a “goodness of fit” measure is used.

Within the described schema particular functions are used to fit the acquired data, for example for the TAC, recovery and S-factor models. The choice of these functions is not discussed, and an obvious fit function from theory may not always be known. In this case an optimal function can be found, and uncertainties reduced by using model selection criteria and model averaging [10, 29, 30].

It is suggested that the major factors affecting uncertainty in the absorbed dose originate from the uncertainty in the delineation of the VOI. Two approaches to determine this uncertainty using statistical and analytical methods are presented. In this example an assumption is made that only a single VOI is applied to all datasets. An alternative approach involves the individual delineation of VOIs for each time point, for which the described methods may need to be varied, taking care to account for any commonalities applied across time points.

Propagation of these uncertainties to derive those further along the dosimetry chain requires the covariance between parameters to be evaluated. An understanding of the variation in VOI counts with VOI uncertainty is challenging as there is no prior knowledge of the count distribution. A method for estimating the count distribution is therefore proposed. However, this approach does not model noise or background counts spilling into the VOI. A more rigorous approach would be to determine a function for change in counts versus volume for the dataset being analysed. However, it is considered that the approach suggested is sufficient since it does not overly complicate the methodology or require additional image processing or analysis, which is not available to the wider nuclear medicine community.

An important feature of the schema is that it can be easily implemented using standard nuclear medicine image processing techniques. This feature is demonstrated in the clinical example in which absorbed dose calculations were performed using a standard image processing workstation and a commercial spreadsheet with curve-fitting software. Clinical implementation of this approach clearly demonstrates how different aspects of the dosimetry calculation can influence uncertainty. Uncertainty pertaining to a smaller lesion is clearly affected by the ability to define precisely the lesion volume and can be significant. For larger organs (such as the liver) volume delineation is less significant and the fit to the TAC begins to dominate. The ability to determine the source of larger uncertainties facilitates optimization of dosimetry protocols.

The clinical example given in the appendix demonstrates the importance of uncertainty in reviewing the significance of results. Figure 12 shows the variation in absorbed doses measured in different treatment cycles. With the presence of uncertainties indicated by error bars, it is possible to determine where a significant difference in delivered absorbed dose occurs. If absorbed dose measurements are to be used to aid future treatment (the goal of MRT dosimetry) it is possible that different treatment strategies could be adopted if the absorbed doses delivered are seen to be constant or decrease with sequential cycles. The uncertainty given in the example demonstrates the utility of the guidance to help identify aspects of the calculations that can be addressed to improve accuracy. It is important to note that the scale of uncertainties should be considered in relation to the range of absorbed doses that are delivered from standard administrations.

Whilst the clinical example demonstrates the use of the schema for SPECT-based dosimetry, the methodology can easily be adapted to suit alternative dosimetry protocols (that is, for multiexponential TAC models, external probe counting or 3D dosimetry). However, variations to the proposed schema should always follow the uncertainty guidelines set out by the GUM.

Uncertainty analysis is important for any measured or calculated parameter, whether physical or biological. Such calculations for MRT are rare [5] and leave room for systematic improvement. With the rapid expansion of MRT and an increase in the number of centres performing dosimetry, it is important for adequate interpretation of the data in clinical practice that measurement uncertainties are quoted alongside absorbed dose values. The application of uncertainty analysis may increase the validity of dosimetry results and may become the basis for quality assurance and quality control. Uncertainty analysis may help identify and reduce errors, aiming at an increased likelihood of observing actual dose–response relationships, which in turn would lead to improved treatment regimens.
